# TOX Outperforms FOXP3, CD4 and GATA3 in Histopathological Diagnosis of Early Mycosis Fungoides

**DOI:** 10.5146/tjpath.2022.01578

**Published:** 2023-01-15

**Authors:** Mona Mostafa Ahmed, Abdelmonem Awad Hegazy, Ahmed Embaby, Esraa Mohammad Nawwar, Salwan Abdelmonem Hegazy, Hanaa M. Ibrahim, Mai Ahmed Gobran

**Affiliations:** Department of Pathology, Zagazig University, Faculty of Medicine, Zagazig, Egypt; Department of Human Anatomy and Embryology, Zagazig University, Faculty of Medicine, Zagazig, Egypt; Department of Medical Biotechnology, Misr University for Science and Technology (MUST), College of Biotechnology, Six of October City, Egypt; Department of Internal Medicine, Faculty of Medicine, Zagazig University, Zagazig, Egypt; Department of Dermatology, Al-Ahrar Teaching Hospital and Zagazig University Hospitals, Zagazig, Egypt; Zagazig University, Faculty of Medicine, Zagazig, Egypt

**Keywords:** Mycosis fungoides, Benign inflammatory dermatoses, Differential histopathological diagnosis, Immunohistochemical markers

## Abstract

***Objective:*** Mycosis fungoides (MF) is the most common type of cutaneous lymphoma. The early stage of MF is a difficult diagnostic case, as it is often confused with many benign inflammatory dermatoses (BID). The study aimed to evaluate the diagnostic utility of TOX, FOXP3, CDD4 and GATA3 in differentiating early stages of MF from histologically overlapping BID lesions.

***Material and Method:*** A retrospective cross-sectional study was performed, in which immunohistochemistry (IHC) was used to evaluate the expression of TOX, FOXP3, CD4 and GATA3 in formalin-fixed paraffin-embedded (FFPE) sections of skin lesions from 30 cases with BID and 30 patients with early-stage MF.

***Results:*** The association between TOX expression and early-stage MF was statistically significant (P <0.001). TOX had the highest sensitivity of 96.77% and accuracy of 85.71% in diagnosis of MF; followed by CD4 with sensitivity of 85.71% and accuracy of 78.95%; and then, GATA3 with sensitivity of 76.7% and finally FOXP3 with sensitivity of 70.0%.

***Conclusion:*** TOX is suggested to be of higher diagnostic value in the early stages of MF than the conventionally used CD4 and other markers examined.

## INTRODUCTION

Mycosis fungoides (MF) is considered the most common type of primary cutaneous T-cell lymphoma (CTCL) (1). It is very difficult to diagnose, especially in the early stages. At these stages, it may be indistinguishable from inflammatory diseases (2). Determining the challenging early stage of MF is critical as it helps in managing cases without delay with favorable subsequent prognosis (3).

Clinically, the disease progresses slowly in three stages including patches, plaques, and tumors (4). MF is histologically characterized by a subcutaneous band-like infiltration with epidermotropic CD4+ neoplastic lymphocytes (5). Therefore, histopathological and immunohistochemical parameters have a pivotal role in its diagnosis. It has been demonstrated that infiltrating epidermotropic neoplastic lymphocytes in MF are predominantly CD4+, with a minority of CD8+ T cells. However, CD4 is also expressed by histiocytes and inflammatory T-lymphocytes, which can be abundant in this context (2) and it must be kept in mind that CD4+ T lymphocytes also take place in inflammatory skin diseases (6).

Transcription factor FOXP3 is used to identify normal regulatory T cells (Tregs) that have been assigned a variety of roles in immune regulation. An increase in normal FOXP3+ Treg cells in dermal infiltrates of MF has been linked to improved prognosis, similar to other indolent lymphomas (7). These cells play a pivotal role in maintaining immune homeostasis and preventing the induction of potent antitumor immune responses (8).

FOXP3 Tregs are found in various lymphomas as well as tumors (9). In a recent study, the majority of malignant T cells in early MF expressed FOXP3 but the expression level on average was much lower than that of non-malignant Tregs (10). Moreover, FOXP3 is positive in the vast majority of T cells of most dermatoses (11).

GATA-binding protein 3 (GATA3), a transcription factor expressed in the epidermis, is essential for the regulation of epidermal differentiation and development of lymphoid cells; GATA3 is required in T helper (Th) cell subset differentiation into Th2 cells (12). GATA3 is expressed on Th2 cells in dermatoses and constitutes the predominant cell in late-stage MF, erythrodermic MF and Sézary syndrome. Immunohistochemical stains for GATA3 is said to be useful in discriminating MF from dermatoses (13,14).

Thymocyte selection-associated high mobility group box factor protein (TOX), which belongs to DNA-binding factors, is involved in the formation of T cells. TOX is upregulated in the thymus during positive selection of CD4+ CD8+ precursors to CD4+ T cells, but it shows a decreased expression in mature CD4+ T cells after leaving the thymus (15). The difficulty in the differential diagnosis between early MF and BIDs may be partially caused by the lack of tumor cell-specific markers. Early studies have reported that TOX is a tumor cell-specific marker of CTCLs including early MF based on immunohistochemical findings, as it is expressed in tumor cells of cutaneous T-cell lymphomas (CTCLs) but barely in inflammatory infiltrate of BIDs (16). TOX is also expressed in infiltrating lymphocytes in BIDs, although the frequency is not high (12,17).

Therefore, in this work we aimed to evaluate the diagnostic utility of common immunohistochemical markers, including TOX, FOXP3, CD4 and GATA3, used in diagnosing early-stage MF in order to avoid misdiagnosis and thus ensure early proper management.

## MATERIALS and METHODS

### Patients

This retrospective cross-sectional study included 30 cases of early MF and 30 cases of BID (15 cases of chronic dermatitis, 10 cases of psoriasis and 5 cases of lichen planus) collected from the archives of the Department of Pathology, Zagazig Faculty of Medicine (2018-2021).

### Ethics

Patients’ consent was obtained; and the research was reviewed and approved by the Institutional Research Board (IRB), (ZU-IRB #6967/-2-6-2021). The research complied with the Helsinki Declaration.

### Study Design

MF was diagnosed by looking at the histologic features of sections stained with hematoxylin and eosin (H&E). All of the included cases of MF had an active lesion in the early stage; and BID cases were also with an active lesion. Pregnant and lactating women and patients with the tumor stage of MF or other types of cutaneous B or T-cell lymphomas were excluded.

The diagnostic feature of MF was the predominance of atypical lymphocytes whose nuclei are larger than those of dermal lymphocytes. The atypical cells had an irregular nuclear border surrounded by a clear halo and were observed in the basal layer or in both stratum basalis and stratum spinosum layers as single cells. Pautrier’s microabscesses were also detected. Clinical findings included the impressions of dermatologists, and the IHC findings (pan T cell markers) were present in the reports.

### IHC Study

Analysis for TOX, Fox and GATA3 was carried out on 5-μm thick archived formalin-fixed paraffin-embedded (FFPE) sections using the Leica Bond III autostainer. The primary antibodies were applied to the slides. They included Rabbit polyclonal anti-TOX antibody, as the optimal primary antibody, at a dilution of 1:150 (Sigma-Aldrich, St. Louis, MO, USA) and GATA3 mouse monoclonal antibody (clone L50-823, 1:600, Cell Marque, Rocklin, California, USA), CD4 (Dako; clone: 4B12, dilution 1:40), CD8 (Dako; clone: C8/144B, dilution 1:50) and FOXP3 mAb (FOXP3 mAb, clone NB100–39002, dilution 1: 400; Novus, St Charles, Missouri, USA). Appropriate negative and positive controls were included. The slides were examined by three pathologists separately.

The IHC evaluation was done by reporting the number of cells with nuclear (FOX, TOX, GATA3) or cytoplasmic-membranous (CD4) staining per 10 high power fields (HPF) in both the epidermis and dermis; and the positive cell percentage was evaluated as Grade 0 if no cells were stained, Grade 1 if < 10%, Grade 2 if 11–50% and Grade 3 if > 50% of cells stained (1,7,14).

### Statistical Analysis

Statistical analysis was assessed using SPSS software, version 11.5 (IBM SPSS Inc., Armonk, NY, USA). The Pearson χ2-test and Fisher exact test were used for comparison of the IHC results between the MF and nonmalignant groups. The p value of ≤0.05 was accepted as statistically significant. Sensitivity, specificity, false negative and positive rates (1−sensitivity), and diagnostic accuracy of each marker were assessed.

## RESULTS

**Figure 1:**
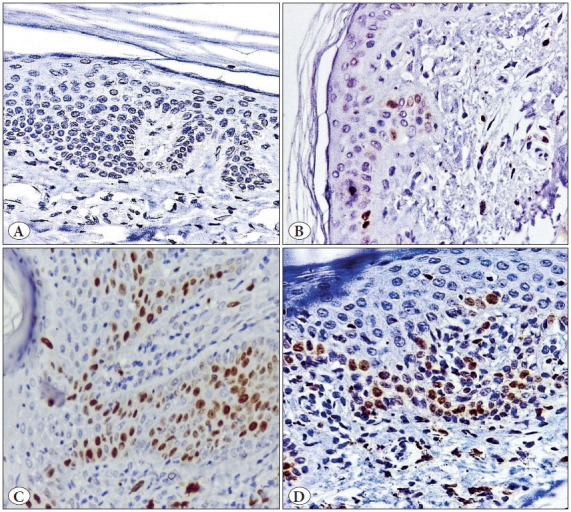
Photomicrographs showing TOX immunoreactivity in different lesions: **A)** A case of chronic dermatitis showing negative TOX immunoreactivity. **B)** A case of chronic dermatitis showing Grade 1 TOX immunoreactivity. **C)** MF case showing Grade 2 positivity of TOX immunoreactivity. **D)** MF case showing Grade 3 TOX immunoreactivity. (Immunoperoxidase stain, x 400)

Sixty cases (30 MF and 30 BID) were included in this study. Evaluation revealed higher grades (2 and 3) of TOX immunohistochemical marker expression in MF cases (33.4% and 60% respectively) than in BID, in which 70% of cases were negative for TOX expression. The results were highly significant (p=0.001) (Table 1; Figure 1).

**Table 1:** Comparing TOX expression between MF and BID.

**Expression**	**Total (n=60)**	**MF**		**BID**		**χ²**	**p-value**
		**n=30**	**%**	**n=30**	**%**		
**TOX expression**						39.8	0.001**
Negative	22	1	3.3	21	70		
G1	6	1	3.3	5	16.7		
G2	13	10	33.4	3	10		
G3	19	18	60.0	1	3.3		
**GATA-3 expression**						14.8	0.002*
Negative	8	7	23.3	1	3.3		
G1	12	2	6.7	10	33.3		
G2	7	1	3.3	6	20.0		
G3	33	20	66.7	13	43.4		
**CD4 expression**						18.3	0.003*
Negative	24	5	16.7	19	63.33		
G1	18	12	40	6	20.0		
G2	4	1	3.3	3	10.0		
G3	14	12	40	2	6.67		
**FOXP3 expression**						14.6	0.002*
Negative	13	4	13.3	9	30.0		
G1	18	11	36.7	7	23.3		
G2	8	0	0.00	8	26.7		
G3	21	15	50.0	6	20.0		

*Statistically highly significant difference (P ≤ 0.001), G*grade

**Figure 2:**
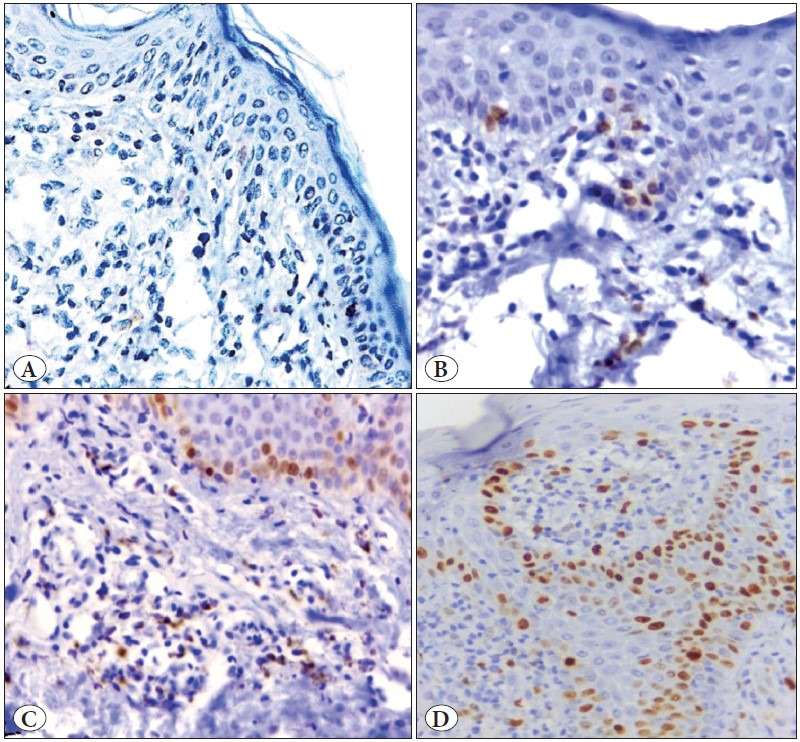
Photomicrographs showing GATA3 immunoreactivity in different lesions. **A)** A case of MF showing negative GATA3 immunoreactivity. **B)** A case of chronic dermatitis showing Grade 1 GATA3 immunoreactivity. **C)** A case of MF showing Grade 2 GATA3 immunoreactivity. **D)** A case of MF showing Grade 3 GATA3 immunoreactivity. (Immunoperoxidase stain, x 400).

GATA3 expression assessment revealed that negative and grade 3 GATA3 expression were higher in MF cases being 23.3% and 66.7%, respectively, compared with BID of 3.3% and 43.4%, respectively; and the results were highly significant (p=0.002). Meanwhile, Grades 1 and 2 GATA3 immunohistochemical expressions were significantly higher in BID (33.3% and 20% respectively) than found in MF cases (6.7% and 3.3%), and the results were highly significant (p=0.002) (Table 1; Figure 2).

**Figure 3:**
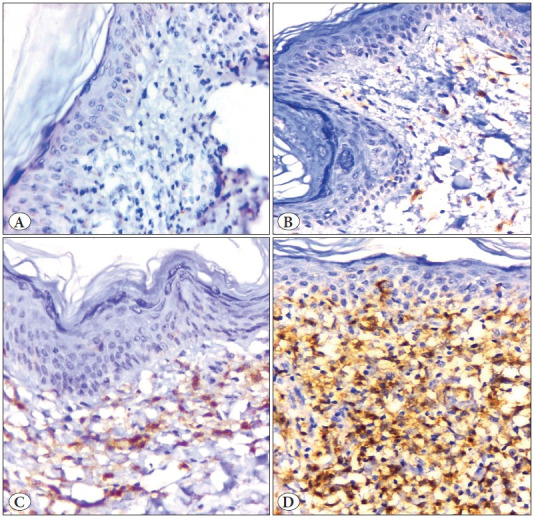
Photomicrographs showing CD4 immunoreactivity in different lesions: **A)** A case of chronic dermatitis with negative CD4 expression. **B)** A case of chronic dermatitis with Grade 1 CD4 expression, showing infiltration of the dermis and the epidermis by a small number of CD4 positive cells. **C)** A case of chronic dermatitis with Grade 2 CD4 expression showing infiltration of the dermis and the epidermis by moderate numbers of CD4 positive cells. **D)** A Case of MF with Grade 3 CD4 expression showing infiltration of the dermis and the basal layer of the epidermis by large numbers of CD4 positive cells. (Immunoperoxidase stain, x 400).

Regarding assessment of CD4 expression, we found that 40% of MF cases showed grade 3 expression while among the BID cases only 6.67% showed it, and the results were significant (p=0.0003). However, negative CD4 immunohistochemical expression was significantly higher in BID (63.3%) than in MF cases (16.7%) (Table 1; Figure 3).

**Figure 4:**
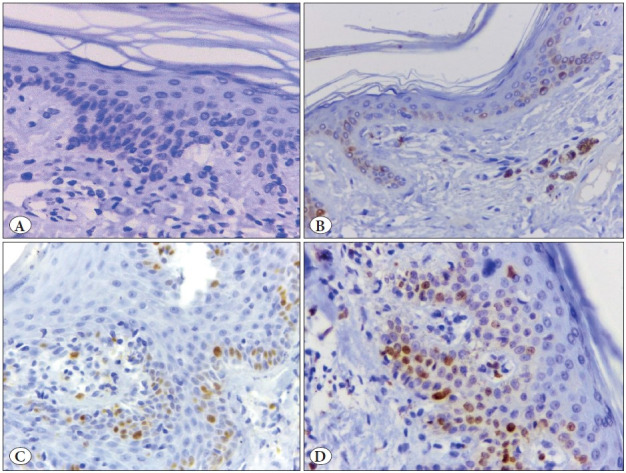
FOX3 immunoreactivity in different lesions: **A)** A case of chronic dermatitis showing negative FOX3 immunoreactivity. **B)** A case of lichen planus showing Grade 1 FOX3 immunoreactivity. **C)** Chronic dermatitis case showing Grade 2 positivity of FOX3 immunoreactivity. **D)** A case of MF showing Grade 3 FOX3 immunoreactivity. (Immunoperoxidase stain, x 400)

Regarding FOXP3 expression, we found that grades 1 and 3 of FOXP3 expression were higher in MF cases (36.7% and 50%, respectively) than in BID cases (23.3% and 20% respectively), and the results were highly significant (p=0.002). However, negative and grade 2 FOXP3 expressions were significantly higher in BID (30.3% and 26.7%, respectively) than in MF cases (13.3% and 0.0% respectively); and the results were highly significant (p=0.002) (Table 1; Figure 4).

**Figure 5:**
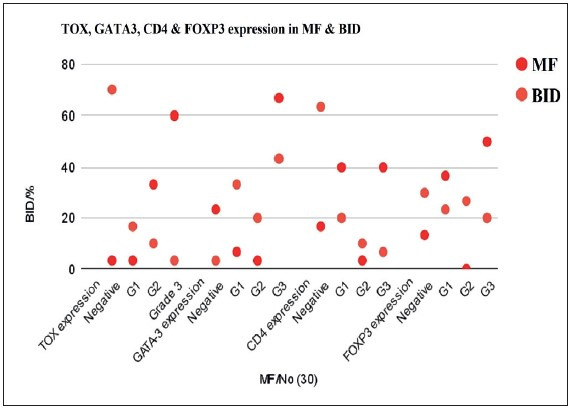
Scatter Chart showing that grade 3 of TOX, GATA3, CD4 and FOXP3 expressions is significantly higher in MF than BID. Grade 1 of GATA3 and TOX expressions is significantly higher in BCID than MF.

The immunohistochemical assessment revealed higher expression (grade 3) of TOX, GATA3, CD4 and FOXP3 in MF than in BID. Negative expressions of TOX, CD4 and FOXP3 were significantly higher in BID than MF cases, but the reverse was true for GATA3 negative expression as it was significantly higher in MF than BID. Moreover, significant differences in other grades between MF and BID were detected (p<0.05) (Table 1; Figure 1, Figure 2, Figure 3, Figure 4, Figure 5).

**Figure 6:**
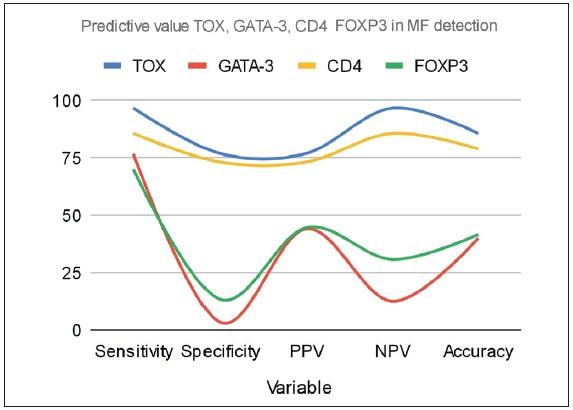
Smooth line chart showing TOX with the highest sensitivity, followed by CD4 with sensitivity and then GATA3 and finally FOXP3.

Investigating the predictive value and sensitivity of TOX, GATA3, CD4 and FOXP3 in MF revealed that TOX had the highest sensitivity (96.77%) followed by CD4 (85.71%), GATA3 (76.7%) and finally FOXP3 (70.0%). Regarding the specificity, TOX had the highest specificity (76.92%) followed by CD4 (73.17%), FOXP3 (13.3%) and finally GATA3 (3.3 %). The marker with highest positive predictive value (PPV) was TOX with 76.92% followed by CD4 (73.17%), FOXP3 (44.7%) and finally GATA3 (44.2%). The marker with highest negative predictive value (NPV) was TOX 96.77% followed by CD4 (85.71%), FOXP3 (30.8%) and finally GATA3 (12.5%). Among the studied markers, TOX had the highest diagnostic accuracy (85.71%) followed by CD4 (78.95%), FOXP3 (41.7%) and GATA3 (40.0%). To sum up, TOX had the highest sensitivity, specificity, PPV, NPV and diagnostic accuracy followed by CD4. FOXP3 was following CD4 as regards specificity, PPV, NPV and diagnostic accuracy but it was less sensitive than GATA3 in distinguishing MF from BID. In other words, GATA3 had the lowest specificity, PPV, NPV and diagnostic accuracy but it was more sensitive than FOXP3 in MF diagnosis than BID** (** Table 2; Figure 6).

**Table 2:** Predictive value of TOX, GATA-3, CD4 and FOXP3 in MF detection

**Variable**	**Sensitivity**	**Specificity**	**PPV**	**NPV**	**Accuracy**
**TOX**	96.77%	76.92%	76.92%	96.77%	85.71%
**GATA-3**	76.7%	3.3%	44.2%	12.5%	40.0%
**CD4**	85.71%	73.17%	73.17%	85.71%	78.95%
**FOXP3**	70.0%	13.3%	44.7%	30.8%	41.7%

## DISCUSSION

The early stage of MF is a challenging clinical condition, as it is often confused with BID. The difficulty in differential diagnosis between early MF and BID may be due in part to a lack of tumor cell-specific markers (18). Finding markers that can distinguish MF from these lesions is a priority. Moreover, the exact etiology of MF is still uncertain, which is why analysis of various molecular markers that may be related to the mechanism of its development may help to determine the pathogenesis of MF. This work investigated the diagnostic potency of TOX, FOXP3, CD4 and GATA3 immunohistochemical expressions in differentiation of MF from the clinically and pathologically overlapping benign lesions.

TOX is one of the proteins that act as a gene expression regulator by changing the structure of chromatin (14). Regarding the development of T cells, TOX is upregulated in the thymus during the positive selection of CD4+CD8+ precursors to CD4+ T cells, but mature CD4+ T cells lose TOX expression and never re-express it again (19). Consistent with previous results of Yu et al. (16) and Huang et al. (20), our study showed that TOX is highly expressed in MF compared to BID. This is because it was expressed in most cases of MF while only observed in 30% of BID. Therefore, TOX could be a potential tool for new therapeutic lines for MF cases.

The FOXP3 gene encodes a transcriptional factor that is vital for the function and development of regulatory Tregs, which are differentiated from naive CD4+ T-helper cells (21). Mutations in FOXP3 induce Treg abnormality and a subsequent defect in immune function, either for inflammatory diseases or tumors (9).

In line with previous reports, the current work revealed that the difference between MF and BID regarding FOXP3 expression was statistically significant (21). Wada et al. (9), found that FOXP3 T cells are less regularly detected in MF than in BID, and its dermal distribution is probably associated with inflammatory components. On the contrary, Fujimura et al. (21) reported that FOXP3 immunoreactivity was significantly weaker in eczematous dermatitis than in MF.

The function of FOXP3 in MF development and progression is quite puzzling. Manso et al. (22) reported that the Tregs number is low at the tumor stage of MF compared to early stages. At the same time, Perelman et al. (23) revealed that FOXP3 expression in the dermis correlated with poor response to treatment. Bhat et al. (24) reported that the worse prognosis of cases of Sézary syndrome with high FOXP3 expression is attributed to its possible role in suppression of immune defenses.

Expression of GATA3 showed a significant difference between MF and BID. This can be explained by the fact that TH1 and not TH2 is the prominent intraepidermal cell in early MF, and GATA3 stains the later one (25).

According to our results, TOX is recommended as a marker for diagnosis of early-stage MF, helping to differentiate it from the benign mimickers. This is because TOX had a high sensitivity (96.77%) and accuracy (85.71%), followed by CD4 for sensitivity (85.71%) and accuracy (78.95%). The same results were obtained by Zhang et al. (15). Morimura et al. (26) reported TOX as a tumor cell-specific marker of CTCLs including early MF based on immunohistochemical findings and TOX was expressed in tumor cells of CTCLs but hardly in inflammatory infiltrates of BID.

The limitations of the study included the relatively small numbers of cases seen at a single medical center. Furthermore, the study did not include all types of BIDs.

## CONCLUSION

Among the immunohistochemical markers examined, TOX is suggested to be the most accurate in diagnosing MF, which is difficult to diagnose especially in its early stages when it is confused with BID. Further future studies including larger numbers of patients are recommended to confirm the diagnostic significance of TOX.

## Conflict of Interest

The authors declare that there is no conflict of interest regarding the publication of this paper.

## Funding

No funding has been received.
